# Catastrophic climate risks should be neither understated nor overstated

**DOI:** 10.1073/pnas.2214347119

**Published:** 2022-10-10

**Authors:** Matthew G. Burgess, Roger Pielke, Justin Ritchie

**Affiliations:** ^a^Center for Social and Environmental Futures, Cooperative Institute for Research in Environmental Sciences, University of Colorado Boulder, Boulder, CO 80309;; ^b^Department of Environmental Studies, University of Colorado Boulder, Boulder, CO 80303;; ^c^Department of Economics, University of Colorado Boulder, Boulder, CO 80302;; ^d^Institute for Resources, Environment and Sustainability, University of British Columbia, Vancouver, BC V6T 1Z4, Canada

Kemp et al. (1) argue that catastrophic climate change scenarios—including societal collapse and human extinction—should be studied explicitly but are currently underexplored. We agree that such scenarios should be studied, and society should prioritize avoiding catastrophic outcomes. However, history also shows risks in overemphasizing the likelihood of calamity. Mindful of this, we argue Kemp et al. understate the degree to which recent scientific and public discourses already prioritize catastrophic climate scenarios.

**Fig. 1.**
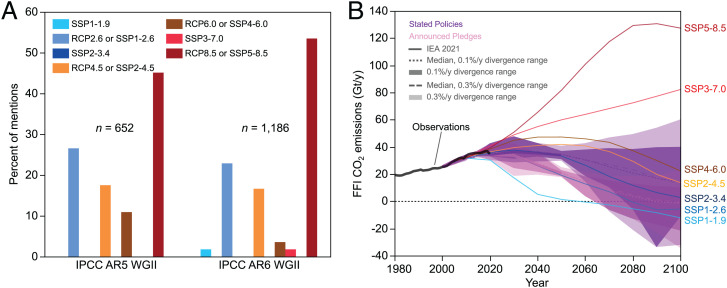
(*A*) Scenario mentions in the IPCC’s Working Group II (Impacts, Adaptation and Vulnerability) contributions to the Fifth (AR5) and Sixth (AR6) Assessment Reports (data from refs. 2 and 3). (*B*) Fossil-fuel-and-industry (FFI) CO_2_ emissions in the seven marker scenarios from AR6, compared to the IEA’s Stated Polices and Announced Pledges scenarios, and to the ranges of all AR6 scenarios having similar projected FFI CO_2_ emissions growth rates from 2005 to 2050 (data from refs. 2 and 4, calculated using the methods of ref. 4).

Kemp et al. (1) note that recent Intergovernmental Panel on Climate Change (IPCC) reports emphasize sub-2 °C scenarios. Simultaneously, IPCC reports also overemphasize catastrophic scenarios, as does broader discourse. For example, the cataclysmic Representative Concentration Pathway 8.5 (RCP8.5) and Shared Socioeconomic Pathway 5-8.5 (SSP5-8.5) scenarios—now widely considered implausible (2)—account for roughly half of the scenario mentions in recent IPCC Assessment Reports’ impacts (Working Group II) sections (Fig. 1*A*), similar to underlying scientific literature (3). The SSP3-7.0 emissions pathway, which Kemp et al. (1) use in their analyses, assumes a world in 2100 heavily reliant on coal and with no climate policy—an implausible future (3, 4). It projects vastly higher emissions than the International Energy Agency (IEA) stated policies scenario, which has continually been revised downward in recent years (4) (Fig. 1*B*).

Could a more plausible high-end emissions scenario, such as SSP2-4.5 (4) (Fig. 1*B*), produce catastrophic climate change? The IPCC associates SSP2-4.5 with a “very likely (5%–95%)” 2.1 °C to 3.5 °C warming range by 2100 (2), under which localized severe impacts are likely (2), and low-probability global catastrophes should still be explored. However, this warming range produces economic damage projections ranging from ∼2 to ∼15% of 2100 global GDP (5). Under the most economically pessimistic [and, possibly, realistic (6)] SSP3, GDP per capita still more than doubles by 2100 in most countries (2, 6). Thus, although highly uncertain, affluence will most likely continue to increase across the vast majority of the world this century, even in relatively pessimistic scenarios.

Overemphasized apocalyptic futures can be used to support despotism and rashness. For example, catastrophic and ultimately inaccurate overpopulation scenarios in the 1960s and 1970s contributed to several countries adopting forced sterilization and abortion programs, including China’s one-child policy, which caused up to 100 million coerced abortions (7), disproportionately of girls. Past and present fascist and neofascist movements frequently use fears of environmental catastrophe to promote eugenics and oppose immigration and aid (8). The Sri Lankan government, concerned about pollution, rashly banned synthetic fertilizers and pesticides in 2021, contributing to an agricultural and economic crisis (9).

Climate catastrophism may be contributing to the youth mental health crisis. In a recent international youth survey, 45% reported thoughts of climate change negatively affecting their daily lives and functioning, and 40% reported being hesitant to have children (10).

In summary, a wide range of climate scenarios should be explored, but, with implausible catastrophic scenarios already a major focus of scientific research, calls for a greater emphasis in this direction risk crowding out a needed focus on more plausible futures.
